# Bioactive Compounds: Multi-Targeting Silver Bullets for Preventing and Treating Breast Cancer

**DOI:** 10.3390/cancers11101563

**Published:** 2019-10-15

**Authors:** Nethaji Muniraj, Sumit Siddharth, Dipali Sharma

**Affiliations:** Department of Oncology, Johns Hopkins University School of Medicine and the Sidney Kimmel Comprehensive Cancer Center at Johns Hopkins, Baltimore, MD 21231, USA; nmunira1@jhmi.edu (N.M.); ssiddha2@jhmi.edu (S.S.)

**Keywords:** bioactive compounds, cancer, withaferin A, honokiol, BITC, resveratrol, curcumin, genistein, EGCG

## Abstract

Each cell in our body is designed with a self-destructive trigger, and if damaged, can happily sacrifice itself for the sake of the body. This process of self-destruction to safeguard the adjacent normal cells is known as programmed cell death or apoptosis. Cancer cells outsmart normal cells and evade apoptosis and it is one of the major hallmarks of cancer. The cardinal quest for anti-cancer drug discovery (bioactive or synthetic compounds) is to be able to re-induce the so called “programmed cell death” in cancer cells. The importance of bioactive compounds as the linchpin of cancer therapeutics is well known as many effective chemotherapeutic drugs such as vincristine, vinblastine, doxorubicin, etoposide and paclitaxel have natural product origins. The present review discusses various bioactive compounds with known anticancer potential, underlying mechanisms by which they induce cell death and their preclinical/clinical development. Most bioactive compounds can concurrently target multiple signaling pathways that are important for cancer cell survival while sparing normal cells hence they can potentially be the silver bullets for targeting cancer growth and metastatic progression.

## 1. Bioactive Compounds in Cancer

Breast cancer is one of the major health concerns for women and the second leading cause of cancer related mortality in the United States (https://www.cdc.gov/cancer/breast/statistics/ index.htm) [1]. In spite of major advancements in novel therapeutic strategies, development of drug resistance is common in all breast cancer subtypes. A significant number of cancers develop resistance towards drugs and relapse accounting for one of the most concerning issue with conventional therapies [2,3]. Conventional therapies mostly fail because of the dysregulation of the balance between cell growth and apoptosis [4]. Although the typical path of development of single target drugs for cancer has produced multiple successful targeted approaches, it has not been able to circumvent the problem of drug resistance and tumor recurrence. Cancer cells exhibit simultaneous activation of multiple cell surface receptors and signaling pathways hence targeting one node typically leads to the activation of alternative pathways. Development of novel anti-cancer drugs with low toxicity and improved efficacy is the need of the hour [5]. 

**Figure 1:**
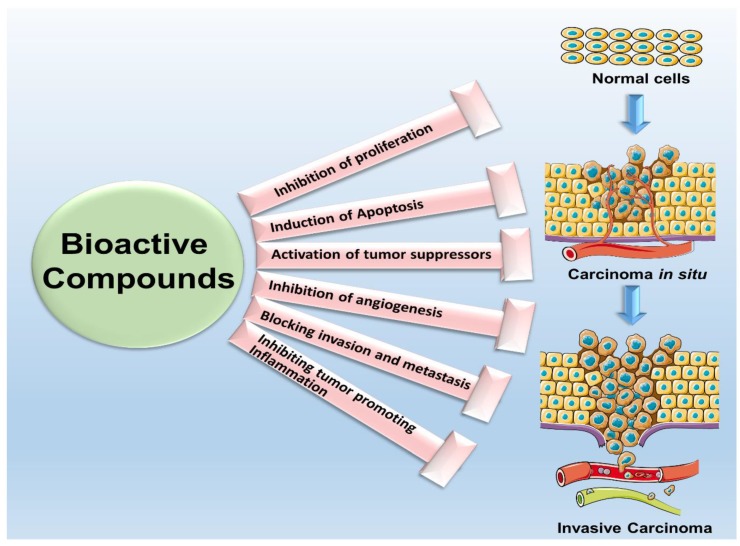
Schematic illustrating the anti-cancer potential of bioactive compounds. Bioactive compounds can inhibit cancer cell proliferation, induce apoptosis, activate multiple tumor suppressors, inhibit angiogenesis, inhibit invasion and migration potential of cancer cells and reduce/inhibit inflammation. Bioactive compounds are multifunctional.

Also known as nutraceuticals, chemicals present in plants and certain foods as natural constituents are termed as bioactive compounds [6]. Bioactive compounds have been used for millennia in Ayurvedic and traditional Chinese therapy for various ailments [7] and have shown efficacy as anti-inflammatory, anti-depressant, anti-microbial as well as anti-cancer agents [5,8]. The discovery of bioactive compounds from plant sources not only established the basis of traditional medicine but is also proving to be an indispensable source of pharmacological agents for modern drug development [9]. Numerous research studies have provided substantial support over the decades for the suitability and effectiveness of several bioactive compounds against cancer [10]. Many successful drugs such as vincristine, vinblastine, doxorubicin, etoposide and paclitaxel have natural product origins. Several bioactive compounds have shown to increase apoptosis of cancer cells through different mechanisms of action [11] and many others have been reported to reduce cancer cell proliferation, induce apoptosis, inhibit invasion and migration and overcome chemo-resistance [5,12]. It has also been shown that several bioactive compounds isolated from medicinal plants delay metastasis and prevent angiogenesis [13,14]. Reports reveal that bioactive compounds affect intracellular signaling associated with carcinogenesis [14,15]. Together, these studies impart solid preclinical support for the clinical development of these bioactive compounds (Figure 1) [16]. 

**Figure 2:**
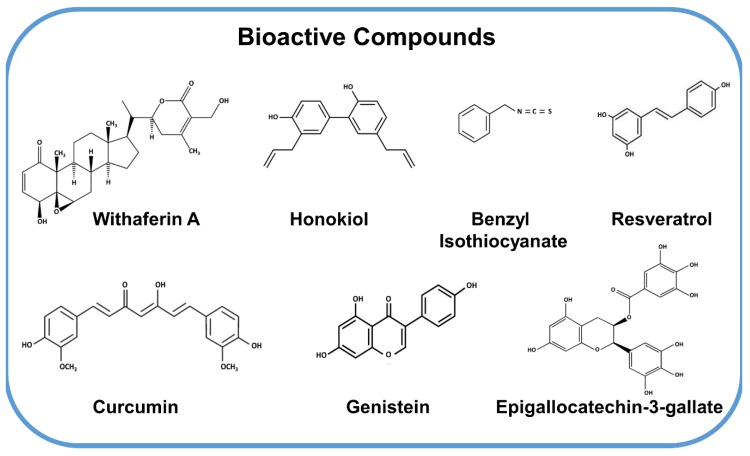
Structures of bioactive compounds. We discuss the anti-cancer potential, underlying molecular mechanisms and clinical development of these select bioactive compounds in this review.

Cancer therapeutics has seen a gradual paradigm shift from monotherapy towards combinational treatment approach and the synergistic effects of multiple bioactive compounds with standard chemotherapy supports this notion. The combination regimen involving bioactive compounds has shown decreased cell proliferation and clonogenicity of breast cancer cells [17]. Evidences suggest that bioactive compounds in combination with chemotherapy increases the efficacy and decreases the toxicity of chemotherapeutic agents [18]. The Dietary guidelines recommend the consumption of whole plant products as chemo preventive agents based on the health advantages observed in epidemiological studies [19] but achieving high levels of phytochemicals in target tissues is always a challenge. One advantage of developing bioactive compounds for cancer prevention and treatment is that these small molecules can be easily taken in effective doses with little or no toxicities. Recent mechanistic studies with various bioactive molecules have shown that most bioactive molecules do not target a single protein or pathway but exert pleiotropic effects concurrently affecting multiple pathways. While outwardly, this may seem like a shortcoming especially in light of the preference for single-target agents but this key characteristic allows bioactive compounds to evade the development of resistance due to the activation of supporting alternative pathways, a problem observed with most single-target drugs [20]. In addition, the effective doses of most bioactive agents do not exert any toxicities or side effects making them ideal preventative and anticancer agents. In this review, we discuss various bioactive compounds (Figure 2), their underlying mechanisms and their efficacy in breast cancer prevention and therapy.

## 2. Withaferin A, a Steroidal Lactone from Ashwagandha

Withaferin A (WFA), a steroidal lactone isolated from *Withania somnifera* commonly known as Ashwagandha, Indian ginseng or winter cherry, is the most potent bioactive compound among the 14 withanolides isolated from this plant. It has been known for its anti-inflammatory, anti-angiogenic, cardioprotective and anti-carcinogenic properties [21,22,23]. WFA modulates cell cycle in breast cancer cells and causes G2 and M phase arrest [24]. *In vivo* and *in vitro* studies from our group and others have revealed that WFA inhibits clonogenicity and induces apoptosis in breast cancer cells [25,26]. WFA administration is correlated with enhanced apoptosis, reduced mammary tumors and decreased pulmonary metastasis in the MMTV (mouse mammary tumor virus)-transgenic model [26]. Reports reveal that WFA-mediated apoptosis is dependent on Bcl-2 [22], ROS [27], Bax and Bak, respectively [28]. Involvement of ROS in WFA-induced cells death has also been shown in breast cancer cells [29,30]. WFA modulates multiple oncogenic signaling pathways in cancer cells to impart its anti-cancer effects. WFA induces anti-tumor effects via STAT3 inhibition in multiple myeloma and neuroblastoma [31]. Sehrawat et al., reported that WFA-mediated apoptosis is associated with the dysregulation of the mitochondrial dynamics in breast cancer cells [32]. In addition, FOXO3 regulates WFA mediated apoptosis in breast cancer cells with its transcriptional target, Bim, which causes reduced cell proliferation and increased tumor mass apoptosis in WFA-treated *in vivo* models [22]. Our recent findings demonstrate that WFA induces apoptosis by downregulating ATP levels and enhancing the activation of AMPK [33]. Importantly, combining 2-deoxyglucose (2-DG) and WFA synergistically enhances apoptosis in breast cancer cells proposing that a combinational regimen may prove more beneficial than either monotherapy [33]. Exhibiting the involvement of downstream effector molecules, Zhang et al., demonstrated that WFA induces caspase 3 and caspase7 leading to apoptosis in MCF-7 cells [34]. Several studies have reported the involvement of tumor suppressor p53 in WFA’s biological functions. Hahm et al., reported that silencing of p53 attenuates WFA-induced apoptosis in breast cancer cells [35]. It has been demonstrated that the growth inhibitory effects of WFA are via induction of p53, p21 and phospho-p38MAPK, as well as by down regulation of ERα, RET and HSF1 [34]. On the contrary, p53-independent effects of WFA have also been demonstrated. WFA-induced apoptosis in breast cancer cells is associated with XIAP, c-IAP-2, and survivin suppression respectively, regardless of p53 or estrogen receptor [28]. Previous study from our lab has shown that WFA treatment activates death receptor 5 (DR5) which leads to efficient growth inhibition [25]. However, WFA has been reported to cause apoptosis in human breast cancer cells through intrinsic and extrinsic mechanism via modulating mitochondrial membrane potential, DNA condensation, cytoplasmic histone-associated DNA fragmentation that causes the degradation cytoskeletal protein and poly (ADP-Ribose) polymerase cleavage [22]. WFA treatment inhibits self-renewal of breast cancer stem cells *in vitro* and *in vivo* [36]. Treating breast cancer cells with a combination of WFA and sulforaphane (SFN) induces apoptosis via upregulation Bax/Bcl-2 ratio and downregulation of HDAC1 expression [37]. Combining WFA with chemotherapy may prove effective as WFA and cisplatin combination effectively inhibits ovarian cancer cells *in vitro* and *in vivo* by eliminating the cancer stem cells [38,39]. Cisplatin and WFA combination treatment has also shown benefit in triple negative breast cancer reducing the cellular proliferation and promoting apoptosis [40]. In summary, the polypharmaceutical effects of WFA holds promise as an anti-cancer compound and warrants further clinical exploration.

## 3. Honokiol, a Polyphenol from Magnolia

In traditional Asian medicine magnolia species have been used for many centuries to treat anxiety, nervous system disorders, fever, gastrointestinal symptoms, and stroke [41,42,43,44,45]. The potential of polyphenols as effective agents against cancer has been acknowledged for the past two decades [46]. Magnolol, one of the bioactive components of *Magnolia officianalis*, is demonstrated to possess inhibitory effects on multiple cancer cell lines [47,48,49,50,51]. Zhou et al., reported that magnolol induces apoptosis and G2/M arrest in MCF-7 cells [52]. Honokiol (HNK) is the major small molecule polyphonic compound derived from *Magnolia grandiflora* and it has been used in traditional Chinese and Japanese medicine. Preclinical *in vitro* and *in vivo* studies reveal anti-tumor, anti-angiogenic, anti-inflammatory and anti-oxidative properties of HNK [41,53,54]. HNK induces apoptosis and inhibits growth in breast and other cancer cells [41,55,56]. Our research group has shown that HNK inhibits breast cancer growth using various *in vitro* and *in vivo* models [54]. Hou et al., demonstrated that HNK induces apoptosis in mouse 4T1 breast cancer model and inhibits cancer cell proliferation *in vitro* and *in vivo* [57]. HNK inhibits nuclear translocation of NFkB and induces TNF-α mediated apoptosis [58]. Tumor microenvironment tend to be hypoxic in nature and HIF-1α helps the cancer cells to adapt to the low oxygen environment [59]. HNK decreases the expression level of HIF-1α and suppresses the hypoxia induced cancer-promoting pathway [60]. Our research group showed that HNK inhibits growth of breast cancer cells by inducing AMP-activated protein kinase in a LKB1-dependent manner [54] and inhibits epithelial-mesenchymal transition and stemness by modulating Stat3/Zeb1/E-Cadherin axis [61]. HNK treatment also effectively inhibits stemness in breast cancer by concurrent activation of tumor suppressor LKB1 and suppression of oncogenic Stat3 signaling [62]. Downregulation of Snail/slug by HNK targets EMT in breast cancer [63]. HNK synergistically induces apoptosis in combination with mTOR inhibitor, rapamycin, in breast cancer cells [64]. HNK modulates TNFα induced Nur77 expression in breast cancer cells [65]. HNK has also shown efficacy in hyperleptinemic obese state where oncogenic hyperactive leptin signaling drives tumor progression. HNK treatment inhibits Wnt1-MTA-1-β-catenin signaling and activates LKB1-miR34a axis leading to the inhibition of breast tumor progression [66,67]. We have shown that HNK impedes cell motility and stem-like breast cancer cell phenotype by decreasing mammosphere formation, OCT4, Nanog, SOX2, and ALDH1, respectively [62]. Studies have suggested that early progenitor cells in breast cancer preserve the capability of self-renewal and differentiation to repopulate the entire tumor [68]. In addition, breast cancer stem cells markers, like aldehyde dehydrogenase (ALDH1), CD24, CD166, CD47, and CD44 are present in abundance in the breast tumor samples [68,69]. HNK attenuates the self-renewal of oral cancer stem cells and reduces the expression of ALDH1 and CD44 cancer stem cell markers [70]. HNK is indeed an attractive bioactive compound with strong anti-cancer potential. Recently, a transdermal approach was developed to facilitate localized delivery of Honokiol that might prove beneficial [71]. Solid preclinical support for honokiol’s anti-cancer potential and an understanding of underlying signaling mechanisms to guide the correlative biomarkers is already present, and now the field is awaiting clinical studies for further development of this promising bioactive compound.

## 4. Benzyl Isothiocyanate, an Isothiocynate from Cruciferous Vegetables

Epidemiological studies have reported that the consumption of cruciferous vegetables is associated with reduced cancer risk [72,73]. Cruciferous vegetables have a high content of glucosinolates and their metabolites, especially isothiocyanates [73,74,75]. Characteristically harboring the N=C=S group, the isothiocyanate class of chemicals is responsible for the well-established medicinal properties of isothiocyanate-rich vegetables and fruits. ITCs naturally occur in abundance in cruciferous vegetables such as broccoli, Brussels sprouts and cauliflower and play an important role in the chemoprevention properties of these vegetables. In a population-based study, broccoli intake inversely correlates with breast cancer in premenopausal women [76,77]. Over the past few decades, research has provided extensive preclinical evidence for the effectiveness of various ITCs against cancer progression [78,79,80]. Benzyl isothiocyanate (BITC), 1-naphthyl isothiocyanate (NITC), phenethyl isothiocyanate (PEITC) and sulforaphane (SFN) are the most significant ITCs that have been extensively researched against various cancers including breast cancer [79]. BITC and SFN reduce cell growth [79], induce apoptosis [81], inhibit spontaneous tumorigenesis in genetically-engineered models and significantly reduce tumor progression in xenografts models [82,83,84]. BITC administration reduces mammary tumor incidence and tumor progression in MMTV-neu mice and tumors in BITC treated group exhibit fewer Ki-67 positive cells and an increase in apoptotic bodies [85]. Yu et al., was first to demonstrate apoptosis induction by ITC in a caspase-3-dependent mechanism [86]. BITC mediated apoptotic-induction in breast cancer cells involves reactive oxygen species generation, increase in pro-apoptotic proteins such as Bax/Bak, reduction in anti-apoptotic proteins such as Bcl-2/Bcl-xL and activation of caspases including caspase 9, caspase 3, and caspase 8 [87]. BITC treatment leads to c-Jun N-terminal kinase (JNK) and p38 mitogen-activated protein kinase (p38 MAPK) activation that plays an important role in BITC’s biological function as their inhibition abrogates BITC-mediated cell death [88]. JNK and p38 MAPK leads to increased Bax translocation from the cytosol to the mitochondria in breast cancer cells [88] and an ectopic expression of the catalytically inactive JNK kinase 2 mutant significantly suppresses the BITC medicated conformational change of Bax [88]. It is interesting to note that BITC can inhibit transcriptional activation of estrogen-responsive genes and disrupt estrogen-estrogen receptor axis in ER-positive breast cancer cells [89]. BITC treated breast cancer cells exhibit FoxO1-mediated autophagy and induces apoptosis via attenuation of mTOR activity *in vitro* and *in vivo* [90]. Inhibition of epithelial-mesenchymal transition (EMT) [91], stemness [92] as well as inhibition of various important oncogenic pathways including Wnt/catenin [93] in response to BITC has been observed. Both p53 dependent and –independent role of BITC has been shown [94,95]. Kim et al., showed that BITC-induced cell death is facilitated by down regulation of X-linked inhibitor of apoptosis (XIAP) in a p53-independent manner [94]. Our research group showed that BITC is effective in breast cancer cells harboring wild type p53 or mutant p53. In the presence of wild type p53, BITC increases p53 phosphorylation and decreases PRAS40 phosphorylation leading to accumulation of active p53 that acts as a transcription factor for tumor suppressor LKB1, and also tethers with LKB1 to upregulate p53-responsive genes. In breast cancer cells harboring mutant p53, BITC dissociates the mutant p53-p73 complex releasing p73 from sequestration. Upon release, p73 activates LKB1 expression and tethers with LKB1 to upregulate p53-responsive genes leading to growth inhibition [95]. Adipocytokine leptin has been shown to turn on an oncogenic signaling cascade and stimulate breast cancer growth in obese state [96,97,98,99,100,101]. Interestingly, BITC treatment can block leptin-induced breast cancer growth by directly inhibiting leptin-mediated Stat3 activation [102]. BITC treated breast cancer cells show decreased tumor progression and lower expression of epithelial-mesenchymal transition (EMT) markers [91]. BITC administration in the MMTV-*neu* mice reduces tumor progression and stemness [103]. Several studies have shown that BITC selectively affects cancer cells and exhibits minimal toxicity against normal cells. BITC effectively suppresses growth of breast cancer cells while normal mammary epithelial cells remain largely unaffected [87,93]. Similarly, human pancreatic cancer cells show increased apoptosis in response to BITC treatment whereas immortalized human pancreatic cells do not respond to BITC [104]. BITC is reported to inhibit growth and induce apoptosis in human oral cancer cells while exhibiting low toxicity to normal cells [105]. These preclinical studies have provided important insights into the anti-cancer efficacy of BITC however clinical validation is still pending.

## 5. Resveratrol, a Polyphenolic Phytoestrogen

Resveratrol (3,5,4’-trihydroxy-*trans*-stilbene) is a non-flavonoid, polyphenolic phytoestrogen majorly present in plants like grapes (*Vitis vinifera*), blueberries, mulberries, soy, pomegranate and peanuts [106,107,108]. Resveratrol was first appreciated in the context of “French Paradox” where resveratrol present in red wine was considered protective against heart disease and obesity in French population despite their high-fat French diet intake [109]. Although it is known today that the beneficial effects of French diet are because of the combination of resveratrol and various other plant-based components and not resveratrol alone, it is still well regarded for its health benefits. Multiple clinical trials have evaluated the efficacy of resveratrol for T2DM, obesity, prediabetes, diabetic neuropathy, NAFLD, fatty liver, brain function, memory, schizophrenia, and Parkinson’s disease [110]. Many trials are currently undergoing to evaluate the benefits of resveratrol in various cancers including colon, colorectal, multiple myeloma and breast cancer [110]. Multiple studies have shown anti-tumor effects of resveratrol in many cancer types [111,112,113,114,115] and its efficacy as a chemopreventive and therapeutic agent is supported by epidemiological and preclinical research [116]. Activation of caspases is an important downstream event in apoptotic induction. Resveratrol has been shown to induce apoptotic death of cancer cells in a caspase-dependent as well as caspase-independent manner [117,118,119]. In estrogen receptor positive breast cancer cells, resveratrol mediates apoptotic cell death via Bcl-2 downregulation that is independent of cytochrome c release and cleavage of caspases 3/8 and PARP [117]. A caspase-3 activation dependent mechanism for apoptotic induction by resveratrol has been shown in estrogen receptor negative breast cancer cells [118]. Modulation of mitochondrial membrane potential, release of cytochrome c, activation of Second Mitochondria-derived Activator of Caspase/direct inhibitor of apoptosis-binding protein with low pI (Smac/DIABLO) and Ca2+-activated protease calpain have also been implicated in resveratrol-mediated apoptotic induction [120]. Interestingly, activation of upstream kinase ERK leads to Bcl-2 suppression in breast cancer cells treated with resveratrol resulting in apoptotic cell death [121]. Resveratrol is also known to inhibit Akt, mammalian target of rapamycin (mTOR), PI3K as well as Wnt/catenin pathway leading to decreased cancer cell growth [122,123]. Another study showed that resveratrol treatment decreases stem cell population in NOD/SCID mice and down-regulates Wnt/β-catenin self–renewal pathway [124]. Examining the involvement of signal transducer and activator of transcription 3 (Stat3) in resveratrol function, Kotha et al., showed that resveratrol inhibits the tyrosine kinase activity of Src leading to the inhibition of Stat3 in breast cancer cells [125]. Resveratrol treatment causes induction of p53, p21 and BRCA in breast cancer cells [126,127,128] via various pathways, one important mechanism being the modulation of epigenetic modulators protein arginine methyltransferase 5 (PRMT5) and enhancer of Zeste homolog 2 (EZH2) and causing changes in histone methylation marks [128]. Owing to its structural resemblance to diethylstilbestrol, a synthetic estrogen, resveratrol has been considered a phytoestrogen that binds to estrogen receptor-α and -β and modulates the expression of ER-responsive genes [129,130]. Multiple studies have evaluated the effectiveness of resveratrol in enhancing the efficacy of chemotherapy or re-sensitizing chemotherapy-resistance cells [131,132,133]. By modulating the SIRT1 and β-catenin axis, resveratrol sensitizes chemotherapy-resistant breast cancer cells to doxorubicin leading to effective inhibition of growth, migration, and EMT [131]. Involvement of miR-122-5p has also been shown in resveratrol-mediated chemo sensitization of Adriamycin-resistant breast cancer cells [132]. Owing to a large number of preclinical studies, the role and importance of resveratrol is already well-established in breast cancer therapeutics and chemoprevention. Multiple ongoing clinical trials will help propel this bioactive compound in clinical arena.

## 6. Curcumin, the Golden Spice

Curcumin is a hydrophobic, polyphenolic constituent of turmeric, the yellow spice extracted from the plant *Curcuma longa*. Curcuma longa grows naturally in Indian subcontinent and South East Asia. The use of turmeric dates to 1900 BCE as a major spice in Indian cuisine and it is commonly used in various homemade and Ayurvedic medicines [134]. Curcumin is the major curcuminoid found in turmeric [134] and has shown to be non-toxic, anti-oxidant, anti-inflammatory and anti-carcinogenic [135]. The contribution of curcumin in science is evident from the establishment of the Curcumin Resource Database (CRDB) incorporating 1186 curcumin analogs, 195 molecular targets, 9075 peer reviewed publications, 489 patents and 176 varieties of *C. longa* [136]. Curcumin inhibits the proliferation of both normal and malignant cells in a non-selective manner, although its apoptotic effect in malignant cells is more profound [137]. Curcumin treatment increases the expression as well as DNA-binding activity of p53 that culminates in modulation of Bax protein and apoptotic induction [138]. Breast cancer cells treated with curcumin show increased levels of pro-apoptotic protein Bax and decreased the expression of anti-apoptotic protein Bcl-2, resulting in an increase in ratio of Bax/Bcl-2 [139,140]. Curcumin derivative (MTH-3) follows extrinsic pathway of apoptosis by upregulating DR5 and FADD and down-regulating anti-apoptotic proteins [141]. Furthermore, MTH-3 shows a substantial rise in levels of CHOP and decreased the levels of IRE1α [141]. These findings indicate that MTH3 causes apoptosis in breast cancer cells via an ER-regulated mechanism through both extrinsic and intrinsic pathways [141]. Curcumin functions via modulating both extrinsic and intrinsic apoptosis pathways. Curcumin inhibits BCL-2 and XIAP resulting in enhanced expression of BAX and BAK members of the Bcl2 family of intrinsic apoptosis regulators [142]. Curcumin mediated cell cycle arrest at the G2/M phase and growth inhibition of breast cancer cells involves reduction in CDC25 and CDC2 expression. Inhibition of Akt phosphorylation, mTOR and Bcl2 along with increased BAX expression and caspase 3 cleavage are also involved in mediating apoptosis in curcumin treated breast cancer cells [143]. Interestingly, curcumin abrogates fatty acid synthase expression and activity in ER negative breast cancer cells exhibiting its potential as a FAS inhibitor [140]. High expression of FAS is observed in many tumor types and these results indicated that curcumin might inhibit multiple tumor types via FAS inhibition. AMPK activation in cancer cells is known to modulate downstream mTOR pathway leading to tumor growth inhibition. Curcumin activates AMPK in breast cancer cells including triple negative breast cancer cells and regulates ERK, p38, and COX-2 [144]. AMPK stimulation in response to curcumin also leads to activation of autophagy pathway and Akt degradation aiding in inhibition of proliferation and migration of breast cancer cells [145]. A study investigating the combined treatment of curcumin and β-interferon (IFN-β)/retinoic acid (RA) showed that curcumin synergistically increases the efficacy of β-interferon (IFN-β)/retinoic acid (RA) in breast cancer cells by upregulating GRIM-19 via Stat3-independent and –dependent pathways [146]. Although antitumor effects of curcumin are well documented in preclinical arena, its negative effect on the efficacy of chemotherapy has also been proposed [147]. Using *in vitro* and *in vivo* breast cancer models, it was shown that curcumin blocks camptothecin, mechlorethamine and doxorubicin induced apoptosis and lowers cyclophosphamide mediated tumor inhibition by inhibiting ROS generation and JNK pathway [147]. However, a plethora of studies showed that curcumin has the potential to function as a chemosensitizer and improves the efficacy of various chemotherapy drugs including bortezomib, paclitaxel, cisplatin, doxorubicin, gemcitabine, 5-FU, oxaliplatin, vincristine, butyrate, celecoxib, vinorelbine, etoposide, sulfinosine, thalidomide, and melphalan in multiple cancer types (reviewed in [148]). Also, curcumin is effective as a radiosensitizer for many cancer types [148]. Aggarwal, et al., revealed that curcumin potentiates paclitaxel cytotoxicity in breast cancer cells and inhibits lung metastasis in xenograft model [149]. Zhang et al., showed that the combination of curcumin and 5-FU increases apoptosis by blocking autophagy and downregulating AKT activity [150]. Combining curcumin and paclitaxel increases the effectiveness of paclitaxel leading to enhanced apoptosis and G2/M cell cycle arrest by increasing caspase-3/7 activity, PARP cleavage and decreasing nuclear factor (NF)-kB transcription factor [151]. Many chemotherapeutic drugs such as doxorubicin are associated with cardiotoxicity which can be successfully overcome by co-administration of curcumin [152]. HO-3867 (3,5-bis(4-fluoro-benzylidene)-1-[(2,2,5,5-tetramethyl-2,5-dihydro-1-hydroxypyrrol-3-yl)methyl]piperidin-4-one), a synthetic analog of curcumin, has been shown to reduce cardiotoxicity but maintain antitumor efficacy when combined with doxorubicin [152]. Mechanistically, curcumin treatment induces the crosstalk between p53 and p300 while alleviating SMAR1-p65NFkB activation leading to resensitization of doxorubicin-resistant cells to doxorubicin [153]. Curcumin treatment also inhibits doxorubicin-induced EMT by inhibiting TGF-β and PI3K/Akt pathways in TNBC and improves the efficacy of doxorubicin [154]. Multidrug resistance can be reversed with a combined treatment of curcumin and doxorubicin using poly(butyl cyanoacrylate) nanoparticles (PBCA-NPs) with co-encapsulated doxorubicin (DOX) and curcumin (CUR) [155]. Co-delivery of curcumin and doxorubicin using core-shell nanoparticles (NPs) with hydrophobic PLLA core loaded with curcumin (Cur) and hydrophilic heparin shell adsorbing doxorubicin (DOX) exhibits promising results for effective breast tumor inhibition [156]. Various research groups have developed nanoparticles containing curcumin alone or in combination with chemotherapies to enhance therapeutic efficacy and bioavailability [157,158,159]. Development of curcumin-loaded solid nanoparticles (Cur-SLN) [157], non-spherical mesoporous silica nanoparticles (MSNAs) [158], and HER2 aptamer-decorated curcumin-loaded human serum albumin nanoparticle (Apt-HSA/CCM NP) [159] are just few examples of nanoparticle approaches. There are multiple other formulations using liposomes, micelles, polymer nanoparticles, nanogels, cyclodextrin complexes, solid lipid nanoparticles (SLN), phytosomes, and gold nanoparticles being developed to further curcumin research [160,161].

## 7. Genistein, a Phytoestrogen from Soy

Genistein (5,7-dihydroxy-3-(4-hydroxyphenyl) chromen-4-one) is an isoflavone phytoestrogen compound present in soybeans and soy food products and is recognized for its beneficial health effects as well as for its chemopreventive and therapeutic properties against multiple cancers [162,163,164,165]. Genistein can effectively bind to estrogen receptors owing to its structural similarity to endogenous 17β-estradiol (E2) [166] raising concerns regarding its potential as a cancer-promoting agent especially for ER-positive breast cancer [167,168]. Additional factors such as genistein intake mode (minimally processed soy foods, MPSFs vs. soy protein isolates) and time of exposure along with individual’s characteristics such as metabolic function, menopausal status, estrogen receptor expression and gene mutations also play an important role in mediating the chemo preventative or cancer-promoting functions of genistein [169]. It is interesting to note that level of soy exposure modulates its biological impact with higher intake of soy associated with lower breast cancer risk [170]. Importantly, clinical studies conducted in Asian population where the soy intake is much higher (5–20 mg/day) show a protective effect of soy on breast cancer risk whereas no correlation is observed in western population with low soy intake (0.8–0.15 mg per day) [170]. *In vitro* and *in vivo* studies support the dose-specific effect of genistein with low doses (≤10 μmol/L) increasing the estrogenic activity while the higher doses (≥10 μmol/L) mediate anti-cancer effects [171,172]. Shim et al., reported that genistein treatment at micromolar concentrations stimulates apoptosis in breast cancer cells via activating calpain, caspase-7 and polymerase (ADP-ribose) in breast cancer cells. Additionally, genistein treated MCF7 cells exhibit enhanced phosphorylation of p38 mitogen-activated protein kinase and apoptosis signaling kinase 1 while ERK ½ phosphorylation remained unaltered [173]. Another study revealed that genistein could block cell proliferation and induce apoptosis by inactivating the IGF-1R-PI3K/Akt pathway and decreasing the expression of Bcl-2/Bax [174]. Genistein treatment reduces the survival of breast cancer stem cells by blocking downstream hedgehog signaling leading to apoptosis [175]. Interestingly, genistein treatment increases the radiosensitivity of breast cancer cells. Breast cancer cells irradiated in the presence of genistein show increased DNA damage and cell cycle inhibition at G2/M phase via activation of ATM, Chk2, Cdc25C and Cdc2 checkpoint pathway. Breast cancer cells cotreated with genistein and radiation also exhibit upregulation of Bax and p73 and downregulation of Bcl2 [176]. Genistein-containing diet does not affect mammary gland proliferation but increases the apoptotic index and expression of PTEN in mammary glands of young adult rats [177]. Treatment with serum from these genistein-fed rats increases apoptosis and PTEN expression in MCF-7 breast cancer cells and, interestingly, silencing of PTEN abrogates the anti-cancer effects of serum from genistein-fed rats [177]. Investigating the chemopreventive potential of genistein, Katdare et al., showed that genistein can modulate cell cycle progression and induce apoptosis in 184-B5/HER breast epithelial cells, a model for comedo-form ductal carcinoma in situ (comedo-DCIS) via downregulation of Her2/neu signaling cascade, upregulation of p16INK4 and BCL2 [178]. This study showed that genistein administration can potentially prevent the progression of DCIS to invasive ductal carcinoma. Invasion and migration are important characteristics of an invasive cancer and genistein blocks invasion and migration of breast cancer cells by inhibiting S-phase kinase-associated protein 2 (Skp2), which is an important kinase frequently upregulated in multiple cancers [179]. Combined treatment with genistein and doxorubicin increases intracellular buildup of doxorubicin and increases efficacy of chemotherapy [180,181]. Inhibition of NK-kB has been implicated in synergistic effect of genistein on various chemotherapy drugs such as cisplatin, docetaxel and doxorubicin [181,182]. Contrasting with the synergistic or additive effect of genistein in potentiating the efficacy of chemotherapy, it has been shown that genistein interferes with the anti-tumor impact of cisplatin in breast cancer cells [172]. Importantly, anti-cisplatin effect of genistein is abrogated in the presence of estrogen in ER-positive breast cancer cells, an effect not observed in ER-negative breast cancer cells [172]. While mechanistic studies are still needed to fully decipher the pathways underlying the pro-tumor vs. anti-tumor effects of genistein, multiple studies have shown how the beneficial effects of genistein can be achieved by carefully titrating its dose in single as well as combination regimens.

## 8. Epigallocatechin-3-Gallate (EGCG), a Green Tea Polyphenol

Epidemiological studies conducted in Japan showed the chemopreventive effects of drinking green tea on breast cancer in women who consumed approximately 10 cups of green tea/day [183]. Many case control and cohort studies have evaluated the effect of green tea on breast cancer and have shown that green tea consumption is associated with a decreased risk of breast cancer [184,185]. Beneficial effects of green tea are associated with catechins class of phytochemicals such as epigallocatechin-3-gallate (EGCG), epicatechingallate (ECG), epigallocatechin (EGC), and epicatechin (EC). EGCG, the ester of epigallocatechin and gallic acid accounts for 40% of total catechins in green tea leaves and is the most important catechin with antioxidant, anti-inflammatory, and anti-carcinogenic potential [186]. Treatment with physiological concentrations of EGCG leads to growth inhibition in breast cancer cells mediated in part by ERα down-regulation, insulin-like growth factor binding protein-2 (IGFBP-2) reduction and p53/p21 upregulation [187]. Nude mice treated with EGCG exhibit inhibition of breast tumor progression and downregulation of multiple key effector molecules including Cyclin D, Cyclin E, CDK 4, CDK 1 and PCNA [188]. Deguchi et al., revealed that breast cancer cells treated with EGCG show increased phosphorylation of JNK/SAPK and p38 which in turn inhibit cdc2 phosphorylation and modulate the expression of Cyclin A, Cyclin Bl, and Cdks, resulting in G2 arrest [189]. Inhibition of Akt along with activation of caspase 3/9 and upregulation of p53 and PTEN leads to apoptotic induction in breast cancer cells treated with EGCG [190,191]. Another study investigating the involvement of Akt in EGCG function showed that EGCG induces growth-inhibition and apoptosis-induction through survivin suppression mediated by blockade of Akt pathway [192]. Induction of apoptosis upon EGCG treatment is associated with enhanced p53 expression, increased release of cytochrome c from mitochondria to cytosol, elevated expression of Apaf-1, and activation of caspase-3 and polymerase (ADP-Ribose) [191]. Also, mouse mammary epithelial cell 4T1 treated with EGCG showed increased p53, Apaf-1, caspase 3 cleavage and high cytochrome C release [193]. A study investigating the dose-dependent effect of EGCG shows that treatment with 50 μM EGCG increases oxidative stress, activation of JNK, and cleavage of caspase 3/9 leading to apoptotic induction while a higher concentration (100–400 μM) causes a necrotic cell death [194]. EGCG also functions through cell membrane-related signaling pathways and alters EGFR, LR, FAS, E-cadherin and β-catenin leading to suppression of cell proliferation, induction of apoptosis through nuclear condensation, and increased caspase-3 activity [195]. EGCG treatment inhibits telomerase activity by decreasing hTERT levels resulting in inhibition of cell growth and induction of apoptosis in MCF-7 breast cancer cells [196]. EGCG mediated growth inhibition and apoptotic induction involves downregulation of miR-25 whose restoration abrogates EGCG-induced apoptosis [197]. Therapeutic efficacy of EGCG has been well established and now efforts are being made to improve its bioavailability, stability and efficacy via utilizing various nanoparticles. Encapsulation of EGCG in the matrix of solid lipid nanoparticles and further conjugation with gastrin releasing peptide receptors (GRPR)-specific peptide not only provides stability to EGCG but also provides specificity towards GRPR overexpressed on breast cancer cells. Treatment with EGCG nanoparticles achieves significantly improved tumor growth inhibition in C57/BL6 mice [198]. EGCG loaded arginyl-glycyl-aspartic acid (RGD)-containing nanostructured lipid carriers (NLC) exhibit improved cytotoxicity and apoptosis in breast cancer cells proposing that EGCG-loaded NLC-RGD may prove beneficial for cancer treatment [199]. FA-NPS-PEG and FA-PEG-NPS also show anti-tumor efficacy in breast cancer cells [200]. Collectively, EGCG shows great potential as a chemopreventive and therapeutic agent.

## 9. Bioactive Compounds Based Anti-Cancer Drugs in Clinical Trials

It is important to note that many successful anti-cancer drugs such as taxol, Vinca alkaloids, combrestatin, epipodophyllotoxin, camptothecin, and their analogues have plant-based origins. Analogues of antimitotic agent paclitaxel/taxol including DHA-paclitaxel, ortataxel, taxol-HMPA polymer, and paclitaxel poliglumex are currently being evaluated in phase I-III clinical trials. Topoisomerase I/II inhibitor-camptothecin-based analogues and podophyllotoxin analogues as well as microtubule destabilizing agents—*Vinca* alkaloids (vinblastine, and vincristine)—analogues are being developed for the clinic (reviewed in [201]). In addition to these drugs with bioactive origins, there are multiple other bioactive compounds that have strong preclinical supporting data underlining their potential as chemopreventive and therapeutic agents against multiple cancers including breast cancer. Clinical development of some of these compounds has begun albeit with a focus on disease conditions other than cancer. (Table 1).

In a randomized, interventional clinical trial for schizophrenia patients, significant benefits were noted upon administration of a standardized extract of *Withania somnifera* (WSE) as compared to placebo control. This study successfully showed that a dose of WSE (1000 mg/day) provided beneficial effects with minimal side effects. Inflammatory markers and cytokine levels were also measured [202] (NCT01793935). Multiple other clinical trials have examined/are examining the clinical benefits of *Withania somnifera* extracts for schizophrenia (WSE 500 mg/day for 12 weeks; NCT03437668); cognitive impairment in elderly subjects (NCT03780621); periodontitis (Ashwagandha capsules 500 mg/day for 30 days; NCT03533972, NCT00010634); endurance exercise performance (NCT03596307), generalized anxiety disorder (NCT01311180); and bipolar disorder (Ashwagandha capsules 250/500 mg/day, NCT00761761). In a multicenter, observational, prospective study including patients with mood disorder, sleep disorder, anxiety or depression, the effects of soy isoflavones in combination with magnolia extract were studied (NCT01805674).

Bioactive compounds are being considered for cancer prevention in high risk population. Phenethyl isothiocyanate was given to the recruits orally 4 times a day for 30 days (NCT00005883) in a phase I trial to examine whether phenethyl isothiocyanate can prevent lung cancer in smokers. It is important to examine the bioavailability of bioactive compounds and a randomized early phase I trial examined bioavailability and absorption of bioactive compounds in broccoli (NCT01743924). In another phase II trial, women with breast cancer/precancerous state (ductal carcinoma in situ and atypical ductal hyperplasia) received broccoli sprout extract three times daily for 2–8 weeks with an aim to examine sulforaphane metabolism, HDAC activity, Ki-67 index and apoptosis (NCT00843167). Results from this trial showed that isothiocyanates, including sulforaphane, were present in urine samples in micromolar concentration along with changes in Ki-67 levels and HDAC activity in tumor samples of the patients in intervention arm.

Resveratrol has been examined for many clinical conditions, such as diabetes, heart failure, pulmonary disease and inflammation (NCT03762096, NCT02245932 and NCT02244879). In a phase I trial, resveratrol was given to colon cancer patients to examine the alteration in Wnt signaling pathway in response to resveratrol (NCT00256334). Resveratrol administration led to inhibition of Wnt pathway in normal colonic mucosa indicating a potential chemopreventive effect of resveratrol in colon cancer [203]. Patients with low-grade GI neuroendocrine tumors were treated with oral resveratrol (5 g/day) to determine the well-tolerated dose of resveratrol and its effect on tumor markers and Notch1 signaling pathway (NCT01476592). Another phase 1 clinical trial determined the safety, pharmacokinetics and modulation of insulin-like growth factor-1 (IGF-1) and IGF-binding protein-3 (IGFBP-3) in response to repeat dosing of resveratrol. Healthy participants received different doses of resveratrol once a day for 29 days. It was observed that 2.5/5 g of resveratrol treatment resulted in decreased IGF-1 and IGFBP-3 in the circulation albeit mild gastrointestinal symptoms were also observed (NCT00098969) [204].

More than 200 clinical trials have been conducted or are ongoing to evaluate the effect of curcumin on various disease conditions (*ClinicalTrials.gov*). An interventional, randomized double-blind study evaluated the efficacy of curcumin for the prevention of radiation-induced dermatitis in breast cancer patients. Breast cancer patients undergoing radiation treatment were given 2.0 g of curcumin thrice/daily for the course of radiation treatment (~4–7 weeks) (NCT01042938). Additional clinical trials (NCT02556632, NCT01246973) have also examined the effect of curcumin-based gel or oral-curcumin on radiation induced dermatitis. A phase II study examined the efficacy of curcumin intervention to reduce NF-kB DNA binding and IL6 levels in chemotherapy-treated breast cancer patients undergoing radiotherapy (NCT01740323). Chemopreventive effects of curcumin were examined in women with obesity and other high-risk factors for breast cancer development and levels of pro-inflammatory biomarkers in plasma and breast adipose tissues were examined (NCT01975363). Multiple clinical trials are currently evaluating the beneficial effects of curcumin for breast cancer prevention, combination-treatment (NCT03072992) and alleviating various breast cancer treatment-related side effects such as aromatase inhibitor-induced joint disease (NCT03865992).

## 10. Conclusions

Despite major advances in the development of novel cancer therapeutics, cancer is still the second leading cause of mortality world-wide with ~9.6 million people succumbing to cancer in 2018. Low and middle-income countries endure a bigger burden with 70% of cancer related deaths occurring in these parts of the world (https://www.who.int/en/news-room/fact-sheets/detail/cancer). Owing to these facts there is an urgent need to develop more affordable and effective therapeutic and preventive strategies to counteract the upsurge of cancer incidences. With the better understanding of molecular subtypes of various cancers, past few years have seen a dramatic increase in “one gene, one drug, one disease” approach to develop novel targeted therapeutic approaches. Although this approach has resulted in many effective drugs, the activation of alternative pathways often circumvents the inhibitory effect of ‘the targeted gene’ leading to the development of resistance. Resistant tumors are often more aggressive than the primary disease and are usually unresponsive to standard treatments owing to the heterogeneity within tumor and the presence of cancer stem cells. Combining various single target drugs is also being evaluated but these cocktails have not proven to be the ‘silver bullets’ either. Nature has performed the best combinatorial chemistry and blessed us with innumerable bioactive compounds that target various hallmarks of cancers simultaneously [205,206]. A plethora of epidemiological studies have shown the beneficial effects of bioactive compounds in various disease states including cancer. Most of these bioactive compounds have been successfully used in traditional medicines such as Ayurvedic, Chinese, Unani, and Homeopathy. As discussed in this review, large numbers of preclinical studies utilizing various in vitro and in vivo model systems have shown the chemopreventive and therapeutic role of various bioactive compounds laying a solid foundation for further clinical development (Table 2). Despite these evidences, clinical studies to evaluate the efficacy of bioactive compounds have been rather limited as the cancer research field has been focusing mainly on synthetic molecules targeting a single gene/protein. It is well known that cancer progression is mediated by an interaction of various signaling pathways but the very fact that bioactive compounds simultaneously modulate various pathways goes against them as the cancer research field remains too focused on single target drugs. Bioactive compounds can be optimal chemopreventive agents since they selectively target cancer cells with no or low toxicity to normal cells, many of them can be included in daily diet, are already part of the food system and are comparatively cheaper.

## Figures and Tables

**Table 1:** Clinical Development of Bioactive Compounds.

Bioactive Compound	Clinical Trials	Phase	ClinicalTrials.Gov Identifier
*Withania somnifera* (Sensoril or Ashwagandha)	A randomized study conducted for schizophrenia	Interventional	NCT01793935
Curcumin and Ashwagandha extract	To study the safety and efficacy of Curcumin and Ashwagandha extract in osteosarcoma.	Phase 1 and Phase 2	NCT00689195
WFA	To evaluate the cognitive abilities in persons with bipolar disorder and to study the residual mood/anxiety symptoms	Phase 3	NCFT00761761
WFA	To study the safety and efficacy of the drug in patients having generalized anxiety disorder	Phase 2	NCT01311180
Resveratrol	Colon cancer patients were randomly given resveratrol at different concentration to study Wnt signaling pathway.	Phase 1	NCT00256334
Resveratrol	To study the effect of Notch signaling in neuroendocrine tumor treated with Resveratrol.	Interventional	NCT01476592
Resveratrol	To study the effect of resveratrol in colorectal cancer and also studied for tolerability, target tissue levels and pharmacodynamics.	Phase 1	NCT00433576
Resveratrol	A randomized trial to determine the disposition and characterize dietary polyphenols in normal and breast cancer patient.	Interventional	NCT03482401
Resveratrol	A randomized double-blind study, resveratrol was given for two weeks in non-diabetic obese subjects.	Phase 2	NCT02247596
Isothiocyanate	A randomized clinical trial, to study phenethyl isothiocyanate in lung cancer patients.	Phase 2	NCT00691132
Isothiocyanate	A randomized phase II study, patients received broccoli sprout extract through oral three times daily for 8 weeks to breast cancer patients, ductal carcinoma in situ and/or atypical ductal hyperplasia	Phase 2	NCT00843167 PMID:26511489 PMID:26329135
Curcumin	A randomized, double-blind, placebo-controlled study of curcumin for the prevention of acute radiation-induced dermatitis during postoperative radiotherapy for breast cancer.	Phase 2	NCT01042938
Curcumin	A randomized A randomized study treating docetaxel alone or together with curcumin in HER2 negative patients with metastatic breast cancer	Phase 2	NCT00852332
Genistein	A randomized study of genistein treatment in high risk of breast cancer patients.	Phase 2	NCT00290758 PMID: 22307566
Genistein	A randomized, phase 1 trial of genistein in preventing breast or endometrial cancer	Phase 1	NCT00099008 PMID:18446090
EGCG	A randomized phase 1 study, to find the effect of catechin extract in hormone negative stage I-III breast cancer patients	Phase 1	NCT00516243
EGCG	A phase 2 trial, to study the effect of green tea extract in breast cancer	Phase 2	NCT00917735 PMID 30926986, 28747487, 27806972, 26701796, 26581683

**Table 2:** Mechanistic Underpinnings of Bioactive Compounds.

Bioactive Compounds	Models	Specific Mechanism of Action	References
Withaferin A (WFA)	Human breast cancer, liver cancer	↑ PARP ↑expression of Bim-S and Bim EL ↓expression of Bcl2 ↑ generation of ROS ↑Bax and Bak ↓expression of XIAP G2/M arrest, ↓ PCNA and ↑apoptosis	[22,27,28,33,37]
Honokiol (HNK)	Human breast cancer	↑G2/M arrest ↑ AMPK	[52,54]
Benzyl Isothiocyanate (BITC)	Human breast cancer	↑ROS ↑Increased caspase 3 ↓mTOR ↓XIAP	[87,88,90,94]
Resveratrol	Human breast cancer	↓Bcl-2, ↓Bcl-XL, ↑Bax ↑Increased caspase 3	[117,118]
Curcumin	Human breast cancer	↓Bcl-2, ↑Bax, ↑Caspase 3, ↑AMPK, ↓AKT ↓pAKT/↓mTOR ↑PARP ↑Caspase3 and ↑caspase 7	[139,143,145,151]
Genistein	Human breast cancer	↑Caspase7, p38MAPK ↓Bcl2 ↑ATM/Chk2/Cdc25C/Cdc2	[173,174,176]
Epigallocatechin-3-gallate (EGCG)	Human breast cancer	↓Cdc ↑Bax, Bcl2, ↑ADP ribose, ↑Caspase 3 ↑hTERT	[189,190,191,196]
